# Gamma frequency entrainment rescues cognitive impairment by decreasing postsynaptic transmission after traumatic brain injury

**DOI:** 10.1111/cns.14096

**Published:** 2023-02-05

**Authors:** Weijie Wang, Xiaotian Zhang, Ruixing He, Shaoxun Li, Dazhao Fang, Cong Pang

**Affiliations:** ^1^ Department of Neurosurgery, Huai'an First People's Hospital Nanjing Medical University Huai'an China

**Keywords:** cognitive impairment, intrinsic excitability, light flicker, long‐term potentiation, postsynaptic transmission, traumatic brain injury

## Abstract

**Introduction:**

The relationship between oscillatory activity in hippocampus and cognitive impairment in traumatic brain injury (TBI) remains unclear. Although TBI decreases gamma oscillations and 40 Hz light flicker improves TBI prognosis, the effects and mechanism of rhythmic flicker on TBI remain unclear.

**Aims:**

In this study, we aimed to explore whether light flicker could reverse cognitive deficits, and further explore its potential mechanisms in TBI mouse model.

**Methods:**

The Morris water maze test (MWM), step‐down test (SDT), and novel object recognition test (NOR) were applied to evaluate the cognitive ability. The local field potential (LFP) recording was applied to measure low gamma reduction of CA1 in hippocampus after TBI. And electrophysiological experiments were applied to explore effects of the gamma frequency entrainment on long‐term potentiation (LTP), postsynaptic transmission, and intrinsic excitability of CA1 pyramidal cells (PCs) in TBI mice. Immunofluorescence staining and western blotting were applied to explore the effects of 40 Hz light flicker on the expression of PSD95 in hippocampus of TBI mice.

**Results:**

We found that 40 Hz light flicker restored low gamma reduction of CA1 in hippocampus after TBI. And 40 Hz, but not random or 80 Hz light flicker, reversed cognitive impairment after TBI in behavioral tests. Moreover, 40 Hz light flicker improved N‐methyl‐D‐aspartate (NMDA) receptor‐dependent LTP (LTP_NMDAR_) and L‐type voltage‐gated calcium channel‐dependent LTP (LTP_L‐VGCC_) after TBI treatment. And gamma frequency entrainment decreased excitatory postsynaptic currents (EPSCs) of CA1 PCs in TBI mice. Our results have illustrated that 40 Hz light flicker could decrease intrinsic excitability of PCs after TBI treatment in mice. Furthermore, 40 Hz light flicker decreased the expression of PSD95 in hippocampus of TBI mice.

**Conclusion:**

These results demonstrated that 40 Hz light flicker rescues cognitive impairment by decreasing postsynaptic transmission in PCs after TBI treatment in mice.

## Graphical abstract

The relationship between oscillatory activity in hippocampus and cognitive impairment in traumatic brain injury (TBI) remains unclear. Although TBI decreases gamma oscillations and 40 Hz light flicker improves TBI prognosis, the effects and mechanism of rhythmic flicker on TBI remain unclear. In this study, we aimed to explore whether light flicker could reverse cognitive deficits, and further explore its potential mechanisms in TBI mouse model. The Morris water maze test (MWM), step‐down test (SDT), and novel object recognition test (NOR) were applied to evaluate the cognitive ability. The local field potential (LFP) recording was applied to measure low gamma reduction of CA1 in hippocampus after TBI. And electrophysiological experiments were applied to explore the effects of the gamma frequency entrainment on long‐term potentiation (LTP), postsynaptic transmission and intrinsic excitability of CA1 pyramidal cells (PCs) in TBI mice. We found that 40 Hz light flicker restored low gamma reduction of CA1 in hippocampus after TBI. And 40 Hz, but not random or 80 Hz light flicker, reversed cognitive impairment after TBI in behavioral tests. Moreover, 40 Hz light flicker improved N‐methyl‐D‐aspartate (NMDA) receptor‐dependent LTP (LTP_NMDAR_) and L‐type voltage‐gated calcium channel‐dependent LTP (LTP_L‐VGCC_) after TBI treatment. And gamma frequency entrainment decreased excitatory postsynaptic currents (EPSCs) of CA1 PCs in TBI mice. Furthermore, our results have illustrated that 40 Hz light flicker could decrease intrinsic excitability of PCs after TBI treatment in mice. These results demonstrated that 40 Hz light flicker rescues cognitive impairment by decreasing postsynaptic transmission in PCs after TBI treatment in mice.

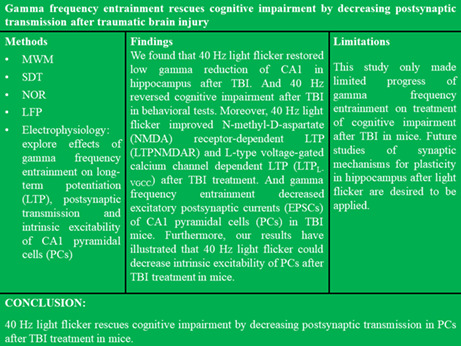

## 1 INTRODUCTION

Oscillatory amplitude, frequency, and its related brain rhythms were involved in some diseases including traumatic brain injury (TBI), Alzheimer's disease, and stroke.^1^, ^2^, ^3^, ^4^ A level of 8–14‐Hz oscillatory activity contributes to a better attenuation in TBI patients during paced auditory serial addition test (PASAT). Although Yang et al., have reported that TBI decreases gamma oscillations and 40 Hz light flicker improves TBI prognosis, the effects and mechanism of rhythmic flicker on TBI remain unclear.

Our previous study has shown that TBI could induce cognitive deficits accompanying the changes in excitability and excitatory transmission in the hippocampus.^5^ The hippocampal gamma frequency entrainment has a close relationship with cognitive ability, and abnormal gamma oscillations were found in neurological disorders.^6^, ^7^ Furthermore, alterations of gamma oscillations after hippocampal local field potential (LFPs) cross‐frequency coupling are related to long‐term potentiation (LTP) reduction in rats.^8^ Only low gamma, rather than theta or high gamma, has been found in a mouse model of hippocampal damage.^9^ However, the role of hippocampal oscillation in the learning and memory after TBI in mice remains unclear. In this study, we aim to explore the effects of light flicker on cognitive ability in TBI mice and explore its potential mechanisms.

Brain oscillations could be manipulated by 40 Hz light flicker in mouse models of Alzheimer's disease and two‐vessel occlusion models in mice and then improve cognitive abilities in those mice.^7^, ^10^ These results suggested that 40 Hz light flicker could reverse cognitive deficits by manipulating brain oscillations. Thus, we aim to explore the potential mechanisms of 40 Hz light flicker on cognitive ability after TBI.

Impairment of hippocampal LTP, reduction of spontaneous synaptic transmission, and intrinsic excitability of CA1 pyramidal cells (PCs) were found in cognitive deficits after TBI.^5^ However, the effects of 40 Hz light flicker on synaptic transmission, LTP, and cognitive ability remain unclear. In this study, we aim to explore rhythmic flicker on cognitive ability and its possible mechanism in TBI mice. Behavioral tests including the Morris water maze (MWM), step‐down test (SDT) and novel object recognition test were applied to detect the cognitive ability, and electrophysiology was used to evaluate the synaptic transmission, intrinsic excitability of PCs and LTP in TBI mice. We found that 40 Hz light flicker can reverse cognitive deficits with decreasing spontaneous postsynaptic transmission, intrinsic excitability of PCs and CA3‐CA1 LTPs in TBI mice.

## 2 METHODS

### 2.1 Animals, TBI mouse model, and visual stimulation

Adult male C57Bl/6 mice (3 months old, 25–28 g) were purchased from Guangdong Provincial Medical Experimental Animal Center. All animal studies were carried out according to protocols approved by the Laboratory Animal Ethics Committee of Nanjing Medical University (permit number: 202255). Meanwhile, all animal studies were carried out under the Guide for the Care and Use of Laboratory Animals (NIH Publication, 2010).

Mouse skull was exposed after being anesthetized using 2.5% isoflurane, and drilled in the middle of the brain. The herringbone sulcus was drilled and kept the dura mater intact during performing operation of TBI. The weight drop of 30 g was set at 3 centimeters above the mouse brain. For the control group, mice were applied to the same procedure without the weight drop injury.

The visual stimulation was performed according to previous study with minor modification.^11^ All mice were placed in a chamber illuminated by a 40 Hz, 80 Hz, or random flicker which was illuminated by a light‐emitting diode (LED) bulb. The six LED lamps were arranged with parallel circuits in order to equally distribute the 40 Hz LED light to the four transparent cages, which housing the four experiment groups at the same time. The 1 h session of visual stimulation was performed 12 h after surgery, and the stimulation was performed twice a day with 12 h intervals that lasted to 25 days post‐TBI (Figure S2A). Moreover, 80 Hz or random flicker visual stimulation was conducted with using the same protocol.

### 2.2 Behavioral experiments

The MWM was performed on days 15–18 after TBI according to the previous study (Figure S2A,B).^12^ A circular pool with 130 cm diameter, 50 cm height was filled with water. A platform with 8 cm diameter was hidden under the surface of the water during the learning phase for 3 days. In the learning phase, all the mice were allowed to find the hidden platform for three consecutive days, 4 trials per day with a 60‐s interval. For each trail, the mice were allowed to start from one of the four quadrants and ended when the mice climbed on the hidden platform by navigation with four visual cues (Figure S2B). The mice were guided to the hidden platform when the mice could not climb on the platform up to 60 s. Memory ability was tested 24 h after the learning phase by removing the platform. Each mouse was allowed to swim freely for 2 min and monitored by using a computer video‐tracking system (Huaibei Zhenghua Biologic Apparatus Facilities Limited Company).

The SDT was carried out 1 day after MWM (Figure S2A,C) according to previous study.^13^ The shock box was a 60 cm × 10 cm × 10 cm plastic box, and the floor of the box was assembled of parallel 0.1 cm‐caliber stainless steel bars with a spacing of 0.5 cm. Four rubber platforms (diameter: 10 cm, height: 4.5 cm) were put in the middle of the shock box. All the mice were placed on the bottom of the shock box and received an electric shock (36 V, AC) on day 19 for 5 min in the training phase. The animals were punished by an electric shock when the mice exposed to stainless steel bars. On day 20, the mice were placed on the rubber platform, and the latency that the time for mice to step down from the rubber platform for the first time was measured, which was applied to measure the memory retention for mice.

The novel object recognition test (NOR) test was performed to detect the memory ability on days 21–24 after TBI (Figure S2A,D). All the mice in this test were subjected to three phases: habituation phase, familiarization phase, and discrimination phase. The habituation phase was performed before the TBI surgery. The mouse was allowed to move for 5 min, and its exploration time on each object was recorded. The distance between the mouse's nose and the object being identified should not exceed 2 cm or the contact with the nose is considered inquiring behavior. In the familiarization phase, the presence of walking around the identified object is not considered to be an inquiry behavior. The discrimination phase was performed 1‐day post‐training and recorded the time of familiar objects (TF) and the time of novelty object (TN). The results were calculated with the following formula: discrimination index (DI) = [(TN − TF)/(TN + TF)].

### 2.3 Intracranial electrode implantation surgeryand LFP recording

Intracranial electrode implantation surgery (IEIS) was performed on day 21 in TBI or control mice (Figure S1A). A cranial window (1.2 mm in diameter, anteroposterior, A/P: 2.0 mm; mediolateral, M/L: 1.5 mm) of the left brain (Figure S1B) was created after the mice were anesthetized with 2.5% isoflurane. A 4‐channel microwire array electrode (35 μm, Stablohm 650, California Fine Wire Co.) was inserted into the CA1 region of hippocampus (Figure S1C). Then the microwire array electrode was fixed to the brain skull bone by using skull screws and reinforced by using dental cement.

Mice were adapted in a 60 cm × 10 cm × 10 cm plastic box for 10 min per day from day 21 to day 25 after TBI until the first LFP recording. The plastic box was cleaned by using 75% ethanol before LFP recording in each mouse. The microwire array electrode was connected to a helium balloon and the mice were allowed to move freely in the plastic box during LFP recording. In order to reduce the impact of mouse movement on the values of theta waves,^14^ the states of approach, exploration, and other low‐speed walking were applied in this study for LFP recording. The microwire array electrode was connected to the OmniPlex Neural Recording Data Acquisition System (Plexon Inc.). Meanwhile, a camera above the plastic box was applied to record the mouse movement during the LFP recording. Moreover, an LED bulb was set in front of the plastic box to perform visual stimulation. The LFPs in the CA1 region were measured for at least 20 min for each mouse. Each mouse received the same pattern of 40 Hz visual stimulation with 2 h break between two mice. A total of six mice for each group were applied for statistical analysis according to the previous study.^7^ Briefly, the sampling rate of LFP signals were set at 1000 Hz with a band‐pass filter set at 05–300 Hz. The NeuroExplorer software (Version 5, Nex Technologies) was used for power spectral analyses. The ratio of power spectral density (PSD) between PSD_exploration_ and PSD_approach_ in theta, low gamma, 40 Hz region, and high gamma were measured.

### 2.4 Electrophysiology

All the mice were sacrificed on day 25 after TBI (Figure S2A), and hippocampal slices (300 μm) were collected by using a Leica vibratome (VT1200S). The hippocampal slices were incubated in artificial cerebrospinal fluid (ACSF) containing 124 mM NaCl, 2.5 mM KCl, 1.25 mM NaH_2_PO_4_, 24 mM NaHCO_3_, 12.5 mM glucose, 5 mM HEPES, 2 mM CaCl_2_·2H_2_O, and 2 mM MgSO_4_·7H_2_O. Titrate pH to 7.4 with 10 N NaOH at 34 ± 0.5 °C for 0.5 h and equilibrated to room temperature for at least 0.5 h. Hippocampal structure was revealed by an upright microscope (ZEISS Axio Examiner).

Patch pipettes (3–5 MΩ) filled with ACSF were applied to record the field excitatory postsynaptic potentials (fEPSPs) by stimulating the Schaffer collateral/commissural (Sch/com). N‐methyl‐D‐aspartate (NMDA) receptor‐dependent LTP (LTP_NMDAR_) was evoked by stimulating with high‐frequency stimulation (100 Hz for 1 s). L‐type voltage‐gated calcium channel‐dependent LTP (LTP_L‐VGCC_) was evoked by four stimulus trains (200 Hz for 0.5 s) with incubation in D‐2‐amino‐5‐phosphonovalerate (D‐APV) (50 μM). LTP_NMDAR_ and LTP_L‐VGCC_ were measured according to responses after stimulation between 0.5 h and 1.5 h. All the data were recorded by using Axon MultiClamp 700B (Molecular Devices) amplifier and analyzed using pClamp 9 (Molecular Devices).

The excitability of the PCs was detected by measuring spontaneous excitatory postsynaptic currents (sEPSCs) and evoked or spontaneous inhibitory postsynaptic currents (sIPSCs), spontaneous APs, evoked APs, resting membrane potential (RMP), and rheobase in an Axopatch‐200B amplifier (Molecular Devices). Patch pipettes (3–5 MΩ) filled with intracellular solution containing 130 mM K‐Gluconate, 4 mM KCl, 10 mM HEPES, 0.3 mM EGTA, 10 mM phosphocreatine‐Na_2_, 4 mM MgATP, and 0.3 mM Na_2_‐GTP were applied to patch clamp recording. Hippocampal structure and CA1 PCs were revealed by an upright microscope (ZEISS Axio Examiner). Responses were low‐pass filtered online at 2 kHz, digitized at 5 kHz. sEPSCs and sIPSCs were measured by holding the membrane potential at −70 mV or −45 mV, respectively, using voltage‐clamp recordings for 3 min. Spontaneous firing rate (SFR) was measured under I = 0 model for 3 min. To measure the excitability of PCs, APs were obtained by injecting a series of depolarizing currents (from −20 pA to 200 pA at a step of 20 pA) using current‐clamp recordings by holding RMP at approximately −70 mV. RMP was measured in the absence of injected current. About 12 cells from three mice per group were analyzed for measuring sEPSCs, sIPSCs, SFR, current‐evoked APs, RMP, and rheobase. The series resistance for all the PCs recorded in this study is within 30 MΩ. The intervals between two recordings were 3 min and allow cells recover from recording.

### 2.5 Immunofluorescence staining

Mice were decapitated, then brain was harvested on day 25 after TBI (Figure S2A). Immunofluorescence staining was conducted as previously described.^15^ In brief, the brain tissues were fixed in 4% paraformaldehyde at 4°C for 48 h and dehydrated in ethanol and embedded in paraffin. The coronal mouse brain sections were obtained. After dewaxing and rehydration, the sections were treated with 0.01 M citrate buffer (pH 6.0) with 0.1% Tween‐20 at 90–95°C for 5 min for antigen retrieval. The sections were incubated at 4 °C overnight with the mouse anti‐NeuN (1:200; Millipore) and rabbit anti‐PSD95 (1:200; Cell Signaling, Inc.). After washing with PBS, the sections were stained with the secondary antibody, anti‐mouse IgG (H + L), F(ab′)2 Fragment (Alexa Fluor® 488 Conjugate) (1:1000; Cell Signaling, Inc.) and anti‐rabbit IgG (H + L), F(ab′)2 Fragment (Alexa Fluor® 594 Conjugate) (1:1000, Cell Signaling, Inc.) for 1 h in the dark at room temperature, and then stained with DAPI (Beyotime Institute of Biotechnology) for 1 min to reveal the nuclei, and analyzed using a laser scanning confocal microscope (Leica). The images were taken using a laser scanning confocal microscope (Leica).

### 2.6 Western blotting

Mice were decapitated, then hippocampus was harvested on day 25 after TBI (Figure S2A). The tissues were homogenized at 4°C by using glass mortar in 50 mM Tris–HCl, pH 7.4, 150 mM NaCl, 10 mM NaF, 1 mM Na_3_VO_4_, 5 mM EDTA, 2 mM benzamidine, and 1 mM phenylmethylsulfonyl fluoride. Primary antibodies against PSD95 (1:1000; Cell Signaling, Inc.) and GAPDH (1:1000; Cell Signaling, Inc.) were diluted using blocking solution to the suggested concentrations and incubated with the membrane at 4°C overnight. After incubation with a secondary antibody for 1 h at room temperature, the membrane was washed. Then the blots were incubated with anti‐mouse IgG conjugated to horseradish peroxidase (1:5000) for 1 h at 37°C. Membrane exposure and data acquisition were achieved by the ImageQuant™ RT ECL System (GE Healthcare).

### 2.7 Statistical analysis

All data were analyzed using GraphPad Prism (Version 7.0) or SPSS (Version 23.0 for windows). The Shapiro–Wilk test for normality was applied to assess data distribution. All the data were presented as the mean ± standard error (SEM). The statistical analysis of escape latency in the learning phase of MWM and DI in the NOR test, etc., by using a 2ANOVA with repeated measures over time followed by Tukey test as shown in the “Results” section. Two‐tailed Student's unpaired t‐test was applied to identify significant groups as shown in figure legends. *p* < 0.05 was considered to indicate statistical significance.

## 3 RESULTS

### 3.1 40 Hz light flicker attenuated neurological deficits of TBI mice

**FIGURE 1:**
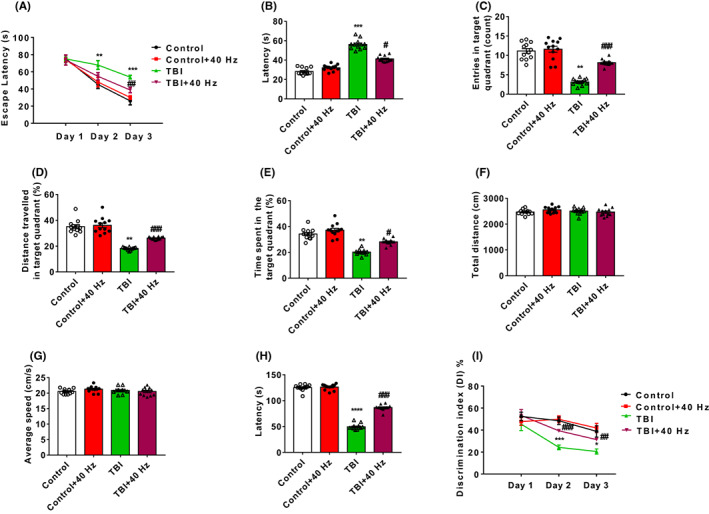
Gamma frequency entrainment attenuated neurological deficits of TBI mice. (A) In the MWM, escape latency was recorded in the learning phase for 3 consecutive days. For measuring the memory ability, latency (B), entries in the target quadrant (C), distance traveled in the target quadrant (D), time spent in the target quadrant (E), total distance (F), and average speed (G) was recorded. (H) In the SDT, latency was measured to evaluate memory retention of mice in all four groups. (I) In the NOR, the DI was obtained to measure working memory of mice in all four groups. *n* = 12 mice for each group. Data were mean ± SEM. Error bars indicated SEM. *p < 0.05, **p < 0.01, and ***p < 0.001 were compared with the control group. ^#^ *p* < 0.05, ^##^ *p* < 0.01, and ^###^ *p* < 0.001 were compared with the TBI group. 2ANOVA with Tukey test was applied in this section. DI, discrimination index; MWM, Morris water maze test; NOR, novel object recognition test; SDT, step‐down test; TBI, traumatic brain injury.

To evaluate the learning and memory abilities of TBI mice, the MWM, SDT, and novel object recognition test (NOR) were performed as depicted in Figure S2A–D. The MWM showed 40 Hz light flicker significantly improved the spatial learning (2ANOVA, *F*(3, 132) = 120.7, *p* < 0.0001, Tukey test *p*_TBI vs. TBI + 40 HZ_ = 0.0064 for day 3, Figure 1A) and memory ability in TBI mice (Figure 1B–E). The SDT test (Figure 1H) and NOR test (2ANOVA, *F*(3, 60) = 12.66, *p* < 0.0001, Figure 1I) have shown that 40 Hz light flicker significantly enhance memory retention or working memory, respectively, after TBI treatment. Random or 80 Hz frequency light flickers did not significantly improve the spatial learning (2ANOVA, *F*(3, 132) = 46.26, *p* < 0.0001, Tukey test *p*_TBI vs. TBI + 40 HZ_ = 0.0008 for day 2, Tukey test *p*_TBI vs. TBI + 40 HZ_ = 0.0082 for day 3, Figure S2E) and memory ability after TBI treatment (Figure S2E–H) in the MWM. The SDT test (Figure S2I) and NOR test (2ANOVA, *F*(3, 60) = 12.66, *p* < 0.0001, Tukey test *p*_TBI vs. TBI + 40 HZ_ >0.05, Figure S2J) have shown that random or 80 Hz frequency light flickers Hz light flicker did not significantly enhance memory retention or working memory, respectively, after TBI treatment.

### 3.2 40 Hz light flicker restored low gamma reduction of CA1 in hippocampus after TBI

**FIGURE 2:**
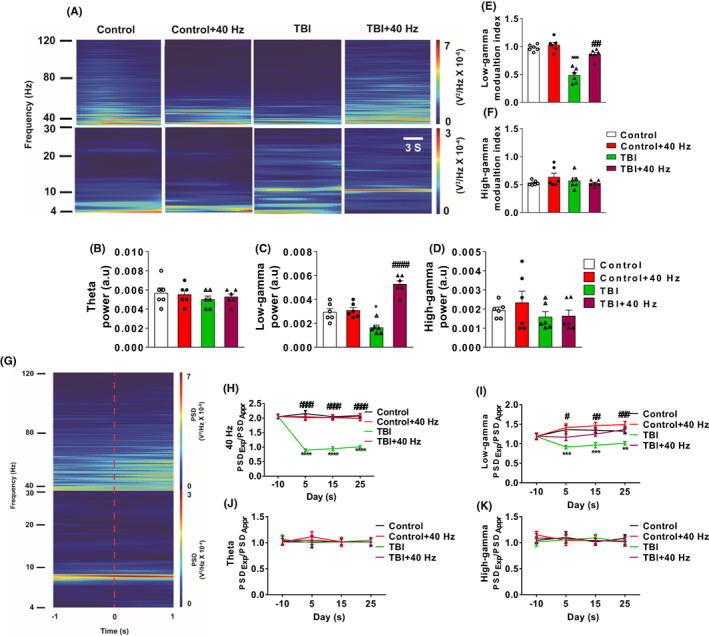
40 Hz light flicker restored low gamma reduction of CA1 in hippocampus after TBI. (A) Typical spectrogram for all the four groups. (B–D) Quantification of the LFP powers of the four groups of mice in the theta, low gamma, and high gamma frequencies. (E) (F) PAC analysis of low gamma and high gamma, respectively, to the theta phase as indicated by the modulation index. (G) Example of moving window spectrogram of hippocampal CA1 LFP time‐locked to the initiation of approach (<0 s) and explore (>0 s) a novel object and the PSD intensity is color‐coded according to the color scale shown on the right. (H–K) The ratio of PSD between Exploration (PSD_Exp_) and Approach (PSD_Appr_) of the four groups of mice at the indicated oscillation frequencies on the *y*‐axis. *n* = 6 mice for each group. Data were mean ± SEM. Error bars indicated SEM. *p < 0.05, **p < 0.01, ***p < 0.001, and ****p < 0.0001 were compared with the control group. ^#^ *p* < 0.05, ^##^ *p* < 0.01, ^###^ *p* < 0.001, and ^####^ *p* < 0.0001 were compared with the TBI group. 2ANOVA with Tukey test was applied in this section. LFP, local field potential; PAC, phase‐amplitude coupling; PSD, power spectral density; TBI, traumatic brain injury.

Previous study has shown that 40 Hz light flicker entrained with hippocampal CA1 low gamma.^7^ Thus, we aim to explore the effects of 40 Hz light flicker on low gamma of CA1 after TBI. Our results have shown that LFP power of CA1 in TBI mice (Figure 2A) was significantly decreased in TBI mice compared to control mice in a range of 30–50 Hz (2ANOVA, *F*(3, 15) = 28.69, *p* < 0.0001, Tukey test *p*_Control vs. TBI_ = 0.0243, Figure 2C). Meanwhile, we found that 4–12 Hz theta (2ANOVA, *F*(3, 15) = 0.4649, *p* = 0.7110, Tukey test *p*_Control vs. TBI_ = 0.6896, *p*_TBI vs. TBI + 40 HZ_ = 0.9698, Figure 2B) and 80–120 Hz high gamma (2ANOVA, *F*(3, 15) = 1.269, *p* = 0.3208, Tukey test *p*_Control vs. TBI_ = 0.8491, *p*_TBI vs. TBI + 40 HZ_ = 0.9996, Figure 2D) were not significantly changed in TBI mice compared to control mice. Moreover, the TBI mice exserted the significant down‐regulation of phase‐amplitude coupling (PAC) between the theta phase and slow gamma amplitude compared to control group mice (2ANOVA, *F*(3, 15) = 31.13, *p* < 0.0001, Tukey test *p*_Control vs. TBI_ < 0.0001, Figure 2E). However, the PAC between theta phase and high gamma did not significant change in TBI mice compared to control group mice (2ANOVA, *F*(3, 15) = 0.9866, *p* = 0.4255, Tukey test *p*_Control vs. TBI_ = 0.9606, *p*_TBI vs. TBI + 40 HZ_ = 0.9363, Figure 2F). Interestingly, we found that 40 Hz light flicker significantly increased CA1 low gamma power in TBI mice compared to control group mice (2ANOVA, *F*(3, 15) = 28.69, *p* < 0.0001, Tukey test *p*_TBI vs. TBI + 40 HZ_ < 0.0001, Figure 2C). Moreover, PAC between the theta phase and slow gamma amplitude was reversed in TBI mice after 40 Hz light flicker treatment (2ANOVA, *F*(3, 15) = 31.13, *p* < 0.0001, Tukey test *p*_TBI vs. TBI + 40 HZ_ = 0.0001, Figure 2E). Taken together, our results have shown that 40 Hz light flicker could reverse the reduction of hippocampal CA1 low gamma in TBI mice.

Our previous results have shown that 40 Hz light flicker significantly enhanced working memory, respectively, after TBI treatment in NOR tests. Meanwhile, the exploration of novel object has a close relationship with 40 Hz low gamma oscillation in CA1 and memory recoding.^16^, ^17^ The LFPs of CA1 were measured in all four groups when the mouse approached or explored the novel object. A spectrogram illustrating an approach (<0 s) or exploration (>0 s) was presented in Figure 2G. Low gamma range of 30–50 Hz (2ANOVA, *F*(3, 80) = 17.20, *p* < 0.0001, *p*_Control vs. TBI_ (day 5) = 0.0006, *p*_Control vs. TBI_ (day 15) = 0.0009, *p*_Control vs. TBI_ (day 25) = 0.007, Figure 2I) or 40 Hz (2ANOVA, *F*(3, 80) = 116.3, *p* < 0.0001, *p*_Control vs. TBI_ (day 5) < 0.0001, *p*_Control vs. TBI_ (day 15) < 0.0001, *p*_Control vs. TBI_ (day 25) < 0.0001, Figure 2H) occurred in the PSD ratio between Exploration/Approach was significantly decreased in TBI mice compared to control group mice. The theta gamma of range 4–12 Hz (2ANOVA, *F*(3, 80) = 0.0702, *p* = 0.9757, *p*_Control vs. TBI_ (day 5) = 0.9659, *p*_Control vs. TBI_ (day 15) = 0.9653, *p*_Control vs. TBI_ (day 25) = 0.9623, *p*_TBI vs. TBI + 40 HZ_ (day 5) = 0.9639, *p*_TBI vs. TBI + 40 HZ_ (day 15) = 0.9982, *p*_TBI vs. TBI + 40 HZ_ (day 25) = 0.9962, Figure 2J) or and the high gamma of range 0–120 Hz (2ANOVA, *F*(3, 80) = 0.0486, *p* = 0.9857, *p*_Control vs. TBI_ (day 5) = 0.9645, *p*_Control vs. TBI_ (day 15) = 0.9922, *p*_Control vs. TBI_ (day 25) = 0.920, *p*_TBI vs. TBI + 40 HZ_ (day 5) = 0.9763, *p*_TBI vs. TBI + 40 HZ_ (day 15) = 0.9672, *p*_TBI vs. TBI + 40 HZ_ (day 25) = 0.9927, Figure 2K) occurred with no significant change in the PSD ratio between Exploration/Approach in all for groups. The TBI‐induced the reduction of 40 Hz (2ANOVA, *F*(3, 80) = 116.3, *p* < 0.0001, *p*_TBI vs. TBI + 40 HZ_ (day 5) < 0.0001, *p*_TBI vs. TBI + 40 HZ_ (day 15) < 0.0001, *p*_TBI vs. TBI + 40 HZ_ (day 25) < 0.0001, Figure 2H) or low gamma (2ANOVA, *F*(3, 80) = 17.20, *p* < 0.0001, *p*_TBI vs. TBI + 40 HZ_ (day 5) = 0.0365, *p*_TBI vs. TBI + 40 HZ_ (day 15) = 0.0068, *p*_TBI vs. TBI + 40 HZ_ (day 25) = 0.0008, Figure 2I) during exploration was significantly reversed by the 40 Hz light flicker. Taken together, our results have illustrated that 40 Hz light flicker reversed TBI‐induced the impairment of CA1 low gamma and theta‐low gamma PAC.

### 3.3 40 Hz light flicker improved CA3‐CA1 LTP of TBI mice

**FIGURE 3:**
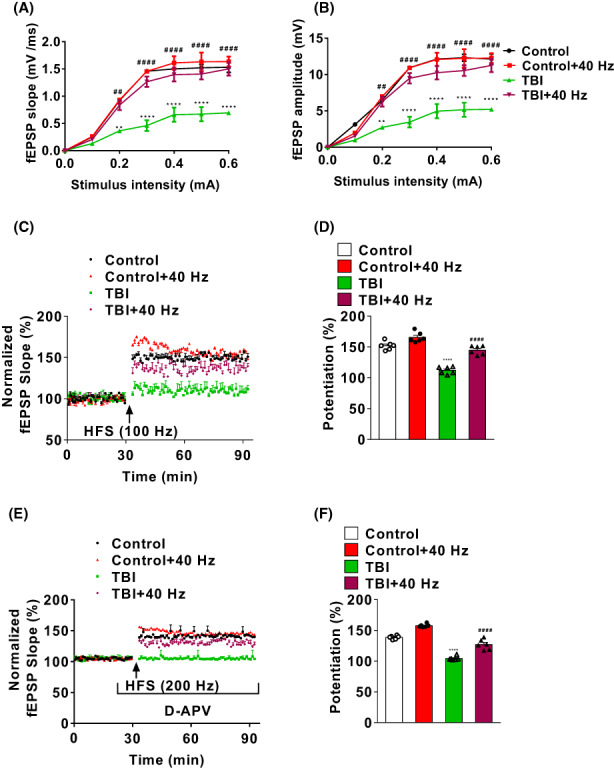
Gamma frequency entrainment improved CA3‐CA1 LTPs of TBI mice. The input/output curve (I/O) of fEPSP slope (A) and amplitudes (B) across stimulation intensities of all four groups of mice (*n* = 6). (C) LTP_NMDAR_ is induced after 100 Hz stimulation in all four groups. (D) The magnitude of LTP_NMDAR_ was measured after high‐frequency stimulation (*n* = 6 mice). (E) LTP_L‐VGCC_ is induced after 200 Hz stimulation and D‐APV treatment in all four groups. (F) The magnitude of LTP_L‐VGCC_ was measured after high‐frequency stimulation. *n* = 6 mice for each group. Data were mean ± SEM. Error bars indicated SEM. **p < 0.01 and ****p < 0.0001 were compared with the control group. ^##^ *p* < 0.01 and ^####^ *p* < 0.0001 were compared with the TBI group. 2ANOVA with Tukey test was applied in this section. LTP, long‐term potentiation; TBI, traumatic brain injury.

Previous studies have shown that theta‐gamma PAC has a close relationship with LTPs and cognitive abilities.^7^, ^17^ The input/output (I/O) curves of field excitatory postsynaptic potentials (fEPSP) slope (2ANOVA, *F*(3, 140) = 104.3, *p* < 0.0001, Tukey test *p*_Control vs. TBI_ < 0.01 and *p*_Control vs. TBI_ < 0.0001, Tukey test *p*_TBI vs. TBI + 40 HZ_ < 0.01 and *p*_TBI vs. TBI + 40 HZ_ < 0.0001, Figure 3A) and amplitude (2ANOVA, *F*(3, 140) = 112, *p* < 0.0001, Tukey test *p*_Control vs. TBI_ < 0.01 and *p*_Control vs. TBI_ < 0.0001, Tukey test *p*_TBI vs. TBI + 40 HZ_ < 0.01 and *p*_TBI vs. TBI + 40 HZ_ < 0.0001, Figure 3B) with a series of increasing stimulation intensities (0.1, 0.2, 0.3, 0.4, 0.5, 0.6 mA) were recorded. Our results have shown that LTP was significantly reduced after TBI treatment, but 40 Hz light flicker improved LTP in TBI mice (Figure 3C, 2ANOVA, *F*(3, 15) = 59.53, *p* < 0.0001, Tukey test *p*_Control vs. TBI_ < 0.0001, *p*_TBI vs. TBI + 40 HZ_ < 0.0001 for Figure 3D). Moreover, D‐2‐amino‐5‐phosphonovalerate (D‐APV), an NMDAR antagonist, was applied to explore the 40 Hz light flicker effects on L‐type voltage‐gated calcium channel‐dependent LTP (LTP_L‐VGCC_). Results have illustrated that LTP_L‐VGCC_ was significantly reduced after TBI treatment and 40 Hz light flicker improved LTP_L‐VGCC_ in TBI mice (Figure 3E, 2ANOVA, *F*(3, 15) = 120.9, *p* < 0.0001, Tukey test *p*_Control vs. TBI_ < 0.0001, *p*_TBI vs. TBI + 40 HZ_ < 0.0001 for Figure 3F). These data have illustrated that 40 Hz light flicker improve postsynaptic LTPs after TBI treatment in mice.

### 3.4 40 Hz light flicker decreased spontaneous synaptic transmission of PCs in TBI mice

**FIGURE 4:**
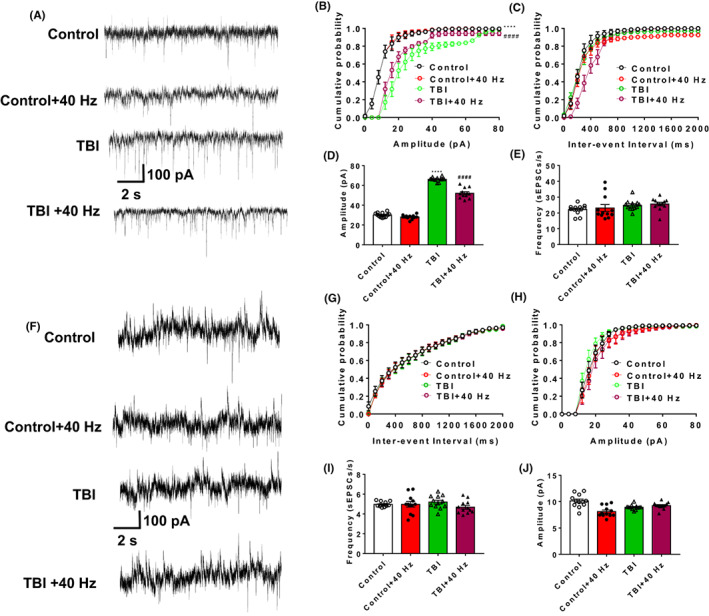
Gamma frequency entrainment enhanced spontaneous synaptic transmission of PCs in TBI mice. (A) Representative current trace of sEPSCs of PCs in all four groups was recorded. Cumulative probability plots of sEPSCs frequency (B) and cumulative probability plots of sEPSCs amplitude (C) in all four groups. Frequency (D) and amplitude (E) of sEPSCs were measured. (F) Representative current traces of sIPSCs of PCs in all four groups were recorded. Cumulative probability plots of sIPSCs frequency (G) and cumulative probability plots of sIPSCs amplitude (H) in all four groups. Frequency (I) and amplitude (J) of sIPSCs were measured. *n* = 12 cells for each group (three mice per group). Data were mean ± SEM. Error bars indicated SEM. ***p < 0.001 and ****p < 0.0001 were compared with the control group, ^#^ *p* < 0.05 and ^####^ *p* < 0.0001 compared with the TBI group. 2ANOVA with Tukey test was applied in this section. PCs, pyramidal cells; sEPSCs, spontaneous excitatory postsynaptic currents; TBI, traumatic brain injury.

Although perirhinal cortex and amygdala are closely related to the SDT or NOR, respectively,^18^, ^19^ previous studies have shown that dorsal hippocampus is involved in the navigation of the MWM.^20^, ^21^ Moreover, our previous results have shown that 40 Hz light flicker improves LTP_NMDAR_ and LTP_L‐VGCC_ after TBI treatment. Thus, CA1 PCs were selected to perform patch clamp recording. The frequency and amplitude of sEPSCs in PCs were up‐regulated in TBI mice compared to control mice, 40 Hz light flicker decreased sEPSCs amplitude (2ANOVA, *F*(3, 924) = 150.3, *p* < 0.0001, Tukey test *p*_Control vs. TBI_ < 0.0001, Tukey test *p*_TBI vs. TBI + 40 HZ_ < 0.0001 for Figures 3B and 4A,B,D) and did not decrease sEPSCs frequency (2ANOVA, *F*(3, 924) = 0.5563, *p* = 0.7698, Tukey test *p*_Control vs. TBI_ = 0.6395, Tukey test *p*_TBI vs. TBI + 40 HZ_ = 0.6538 for Figures 3C and 4A,C,E) in TBI mice. Interestingly, all four groups of mice had no difference in the frequency (2ANOVA, *F*(3, 924) = 0.5673, *p* = 0.6367 for Figures 3G and 4F,G,I) and amplitude of sIPSCs (2ANOVA, *F*(3, 924) = 0.5673, *p* = 5.876 for Figures 3H and 4F,H,J). These data may illustrate that 40 Hz light flicker decrease excitatory postsynaptic currents (EPSCs) of PCs in TBI mice.

### 3.5 Effects of 40 Hz light flicker on intrinsic excitability of PCs in TBI mice

**FIGURE 5:**
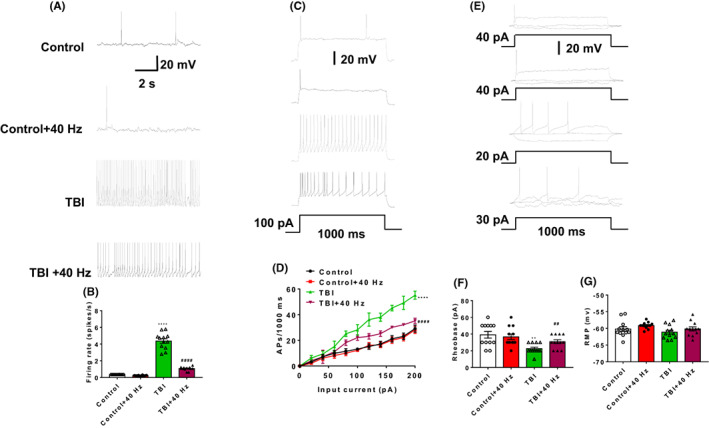
Effects of gamma frequency entrainment on intrinsic excitability of PCs in TBI mice. (A) Representative traces of spontaneous APs in PCs were recorded in all four groups. (B) Firing rate of spontaneous action potentials (APs) in PCs were recorded in all four groups. (C) Representative traces of evoked APs in PCs were presented. (D) Number of evoked APs against the injected current in PCs were measured in all four groups. (E) Representative current clamp trace of action potential elicited at rheobase in PCs were measured in all four groups. (F) Rheobase in PCs was measured in all four groups. (G) RMP in PCs was measured in all four groups. *n* = 12 cells for each group (three mice per group). Data were mean ± SEM. Error bars indicated SEM. ***p < 0.001 was compared with the control group, ^##^ *p* < 0.01 and ^###^ *p* < 0.001 were compared with the TBI group. 2ANOVA with Tukey test was applied in this section. RMP, resting membrane potential; PCs, pyramidal cells; TBI, traumatic brain injury.

To explore the role of 40 Hz light flicker on intrinsic excitability of PCs in TBI mice, spontaneous APs, current‐evoked APs, and rheobase were measured. The number of spontaneous APs (2ANOVA, *F*(3, 33) = 1014, *p* < 0.0001, Tukey test *p*_Control vs. TBI_ < 0.0001, Tukey test *p*_TBI vs. TBI + 40 HZ_ < 0.0001 for Figure 5A,B) and current‐evoked APs (2ANOVA, *F*(3, 484) = 180.9, *p* < 0.0001, Tukey test *p*_Control vs. TBI_ < 0.0001, Tukey test *p*_TBI vs. TBI + 40 HZ_ < 0.0001 for Figure 5C,D) were significantly decreased after 40 Hz light flicker in TBI mice. Moreover, 40 Hz light flicker significantly increased rheobase in TBI mice (Figure 5E, 2ANOVA, *F*(3, 33) = 6.996, *p* = 0.0009, Tukey test *p*_Control vs. TBI_ = 0.001, Tukey test *p*_TBI vs. TBI + 40 HZ_ = 0.0057 for Figure 5F). All four groups of mice had no difference in the resting membrane potential (RMP) (2ANOVA, *F*(3, 33) = 2.443, *p* = 0.0816, Tukey test *p*_Control vs. TBI_ = 0.5949, Tukey test *p*_TBI vs. TBI + 40 HZ_ = 0.5622 for Figure 5G). These data may illustrate that 40 Hz light flicker decrease intrinsic excitability of PCs in TBI mice.

### 3.6 Effects of 40 Hz light flicker on the expression of PSD95 in hippocampus of TBI mice

**FIGURE 6:**
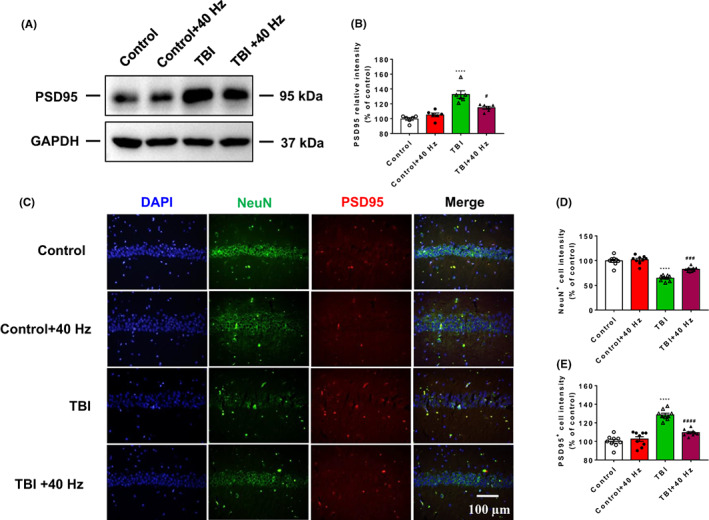
Effects of 40 Hz light flicker on the expression of PSD95 in hippocampus of TBI mice. Western blot analysis showed the levels of PSD95 in hippocampus (A, B) (six mice per group). Immunofluorescence staining showed the levels of NeuN and PSD95 in CA1 of hippocampus in all four groups (C–E) (*n* = 9 sections from three mice for each group). Scale = 100 μm. Data were mean ± SEM. Error bars indicated SEM. ****p < 0.0001 was compared with the control group, ^#^ *p* < 0.05, ^###^ *p* < 0.001 and ^####^ *p* < 0.0001 were compared with the TBI group. 2ANOVA with Tukey test was applied in this section. TBI, traumatic brain injury.

To explore the role of 40 Hz light flicker on the expression of PSD95 in hippocampus of TBI mice, immunofluorescence staining and western blotting were applied. Our results revealed that TBI significantly increased the expression of PSD95 in hippocampus. Moreover, 40 Hz light flicker significantly decreased the expression of PSD95 in hippocampus of TBI mice (Figure 6A,B, 2ANOVA, *F*(3, 15) = 16.75, *p* < 0.0001, Tukey test *p*_Control vs. TBI_ < 0.0001, Tukey test *p*_TBI vs. TBI + 40 HZ_ = 0.0134 for Figure 6B). Results of immunofluorescence staining have shown that TBI significantly decreased the expression of NeuN in the CA1 region of hippocampus. Moreover, 40 Hz light flicker significantly increased the expression of NeuN in the CA1 region of hippocampus (Figure 6C,D, 2ANOVA, *F*(3, 24) = 44.55, *p* < 0.0001, Tukey test *p*_Control vs. TBI_ < 0.0001, Tukey test *p*_TBI vs. TBI + 40 HZ_ = 0.0003 for Figure 6D). Furthermore, results of immunofluorescence staining have shown that TBI significantly increased the expression of PSD95 in the CA1 region of hippocampus. Moreover, 40 Hz light flicker significantly decreased the expression of PSD95 in the CA1 region of hippocampus (Figure 6C,E, 2ANOVA, *F*(3, 24) = 67.8, *p* < 0.0001, Tukey test *p*_Control vs. TBI_ < 0.0001, Tukey test *p*_TBI vs. TBI + 40 HZ_ < 0.0001 for Figure 6E). These data may illustrate that 40 Hz light flicker decreased the expression of PSD95 in hippocampus of TBI mice.

## 4 DISCUSSION

In this study, results have shown that 40 Hz light flicker reverses cognitive impairment in the MWM, SDT and novel object recognition test (NOR) tests after TBI in mice. Moreover, 40 Hz light flicker restored low gamma reduction of CA1 in hippocampus after TBI. Moreover, we found that 40 Hz light flicker improves NMDA receptor‐dependent LTP (LTP_NMDAR_) and L‐type voltage‐gated calcium channel‐dependent LTP (LTP_L‐VGCC_) after TBI treatment. And gamma frequency entrainment decreased amplitude of EPSCs of CA1 PCs in TBI mice. Furthermore, our results have illustrated that 40 Hz light flicker could decrease intrinsic excitability of PCs after TBI treatment in mice. Moreover, 40 Hz light flicker decreased the expression of PSD95 in hippocampus of TBI mice. Thus, we suspect that gamma frequency entrainment could reverse cognitive deficits through the mechanisms involving postsynaptic transmission.

Studies have shown that hippocampal gamma oscillations could be modulated by 40 Hz light flicker and then involved in the process of cognitive ability.^10^, ^11^, ^22^, ^23^ Previous study found that gamma oscillations of the injury side after TBI in rats is decreased, and 40 Hz light flicker could attenuate the down‐regulation of gamma oscillation after TBI.^24^ Thus, we suspected that gamma oscillations, which could be affected by 40 Hz light flicker, is manipulated and then involved in the process of cognitive ability after TBI treatment in mice. In this study, our results have shown that 40 Hz light flicker restored low gamma reduction of CA1 in hippocampus after TBI. Moreover, our results illustrated that 40 Hz light flicker could rescue cognitive impairment by using MWM, SDT, and NOR tests after TBI in mice. However, 80 Hz or random light flicker did not significantly enhance cognitive impairment induced by TBI in mice. Thus, we applied 40 Hz light flicker to explore the role of hippocampal oscillation in cognitive ability after TBI in mice.

Results of previous studies have illustrated that NMDA receptor play a key role in cognitive ability by affecting neuron survival in neurodegenerative disorders.^25^, ^26^, ^27^ Moreover, L‐type voltage‐gated calcium channel (L‐VGCC) activity in postsynaptic neurons is involved in cognitive process by perturbing calcium homeostasis and then transferring survival signaling to its downstream neurons.^28^ Inhibiting LTP_L‐VGCC_ in the hippocampus induced the neuronal death of CA1 region in a two‐vessel occlusion (2VO) mice model.^7^, ^29^, ^30^ Our study has shown that flickering light of 40 Hz enhanced both LTP_NMDAR_ and LTP_L‐VGCC_ in the hippocampus of TBI mice. Thus, we suspected that 40 Hz light flicker enhances postsynaptic strength in hippocampus and then improves cognitive ability in TBI mice.

The activation of NMDAR was reported to be involved in the LTP_NMDAR_ induction and then enhanced EPSCs.^31^, ^32^ The enhancement of LTP_NMDAR_ induced the alteration of NMDAR EPSCs on time course,^33^ and LTP coupled with EPSCs in hippocampus was found to be involved in the spatial working memory.^32^ In hippocampus, EPSCs be evoked by NMDARs and contributed to the alteration of calcium homeostasis and synaptic activity after high‐frequency stimulation (HFS).^34^ Interestingly, 40 Hz light flicker decreased amplitude of sEPSCs, decreased the number of spontaneous APs, current‐evoked APs of PCs without changes in frequency of sEPSCs in TBI mice. Previous study has found that increasing sEPSC amplitude without changes in sEPSC frequency has been related with postsynaptic mechanisms.^4^ Moreover, gamma frequency entrainment increased rheobase and had no effects on resting membrane potential (RMP) of PCs in TBI mice. Moreover, 40 Hz light flicker decreased the expression of PSD95 in hippocampus of TBI mice. Thus, we suspected that postsynaptic mechanisms may be response for cognitive enhancement after gamma frequency entrainment in TBI mice.

This study only made limited progress of gamma frequency entrainment on treatment of cognitive impairment after TBI in mice. Future studies of synaptic mechanisms for plasticity in hippocampus after light flicker are desired to be applied. Although one limitation of this study is that all the mice were applied to electrophysiological experiments after the MWM, SDT, and NOR, principle of the three R's (replacement, reduction, and refinement) was applied in this study.

## 5 CONCLUSION

40 Hz light flicker rescues cognitive impairment by enhancing postsynaptic transmission in CA1 PCs after TBI treatment in mice, which may become a promising therapeutic strategy in clinic.

## AUTHOR CONTRIBUTIONS

WJW designed and supervised the study. WJW, XTZ, RXH, SXL, DZF, and CP performed the experiments and collected the data. WJW, XTZ, and RXH drafted and finalized the manuscript. WJW and XTZ edited the manuscript. All authors read and approved the final manuscript.

## FUNDING INFORMATION

This project was supported by Key Research and Development Plan Projects (Social Development) in Huai'an city of Jiangsu Province (Nos. HAS2015018 and ZD2021051) for Lianshu Ding and the Natural Science Research Program in Huai'an city of Jiangsu Province (HAB201726) for Weijie Wang.

## CONFLICT OF INTEREST STATEMENT

All authors declare no conflict of interests.

## Supporting information


Data S1.

Figures S1–S2.

## Data Availability

The data that support the findings of this study are available from the corresponding author upon reasonable request.
