# Supplementary figures and images for: Gamma frequency entrainment rescues cognitive impairment by decreasing postsynaptic transmission after traumatic brain injury
Source: CNS Neurosci Ther. 2023 Feb 5;29(4):1142–53. doi: 10.1111/cns.14096 (PMC10018095; doi:10.1111/cns.14096)

PSD95-Full unedited gel blot for Figure 6A

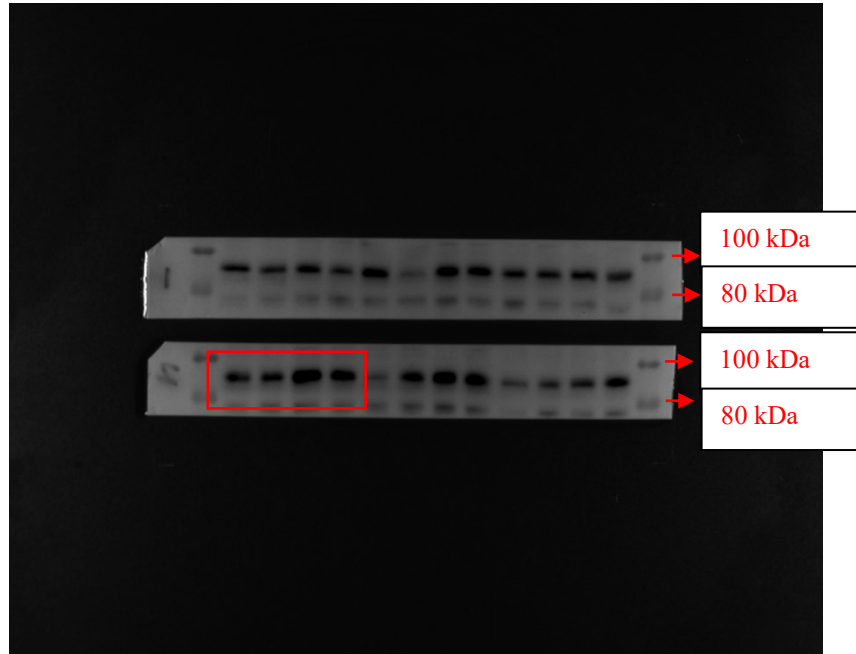

GAPDH-Full unedited gel blotfor Figure 6A

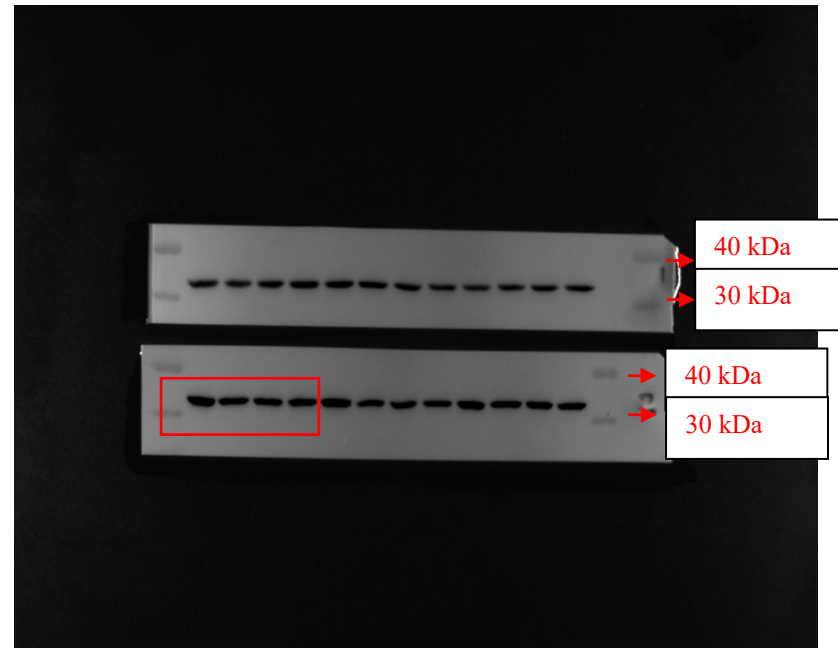

Supplement: Supplementary file 1 — Data S1. [file CNS-29-1142-s002.pdf]
