# Supplementary material for: Gamma frequency entrainment rescues cognitive impairment by decreasing postsynaptic transmission after traumatic brain injury
Source: CNS Neurosci Ther. 2023 Feb 5;29(4):1142–53. doi: 10.1111/cns.14096 (PMC10018095; doi:10.1111/cns.14096)
Supplement: Supplementary file 2 — Figures S1–S2. [file CNS-29-1142-s001.docx]

**Supplementary Figures:**

**Fig. S1. Effects of light flicker on local field potentials (LFPs) in TBI mice. A** Schedule of TBI, intracranial electrode implantation surgery (IEIS), light flicker treatment and LFP recording. A multielectrode array was dorsally implanted into the CA1 region of hippocampus 21 days after TBI. LFP recoding was performed on day 25 after TBI on free-moving mice (**B, C**).

**Fig. S2. Gamma frequency entrainment attenuated neurological deficits of TBI mice. A** Schedule of TBI, light flicker treatment and test orders. **B** In the MWM, escape latency was recorded in the learning phase for 3 consecutive days. For measuring the memory ability, latency (**C**), entries in the target quadrant (**D**), time spent in the target quadrant (**E**) were recorded. **F** In the SDT, latency was measured to evaluate the working memory. **G** In the NOR, the DI was obtained to measure working memory in all four groups. n = 12 mice for each group. Data were mean ± SEM. Error bars indicated S.E.M. ^*^*P*<0.05, ^**^*P*<0.01, and ^***^*P*<0.001 were compared with TBI group. 2ANOVA with Tukey test was applied in this section.
